# Transcriptome changes during fruit development and ripening of sweet orange (*Citrus sinensis*)

**DOI:** 10.1186/1471-2164-13-10

**Published:** 2012-01-10

**Authors:** Keqin Yu, Qiang Xu, Xinlei Da, Fei Guo, Yuduan Ding, Xiuxin Deng

**Affiliations:** 1 Key Laboratory of Horticultural Plant Biology of Ministry of Education, Huazhong Agricultural University, Wuhan 430070, China; 2 National Key Laboratory of Crop Genetic Improvement, Huazhong Agricultural University, Wuhan 430070, China

## Abstract

**Background:**

The transcriptome of the fruit pulp of the sweet orange variety Anliu (WT) and that of its red fleshed mutant Hong Anliu (MT) were compared to understand the dynamics and differential expression of genes expressed during fruit development and ripening.

**Results:**

The transcriptomes of WT and MT were sampled at four developmental stages using an Illumina sequencing platform. A total of 19,440 and 18,829 genes were detected in MT and WT, respectively. Hierarchical clustering analysis revealed 24 expression patterns for the set of all genes detected, of which 20 were in common between MT and WT. Over 89% of the genes showed differential expression during fruit development and ripening in the WT. Functional categorization of the differentially expressed genes revealed that cell wall biosynthesis, carbohydrate and citric acid metabolism, carotenoid metabolism, and the response to stress were the most differentially regulated processes occurring during fruit development and ripening.

**Conclusion:**

A description of the transcriptomic changes occurring during fruit development and ripening was obtained in sweet orange, along with a dynamic view of the gene expression differences between the wild type and a red fleshed mutant.

## Background

The typical course of fruit development involves expansion, sweetening and increasing pigmentation [1]. From the consumers' point of view, the appearance, texture and taste of the fruit are all of high importance. These properties involve attaining a suitable composition of sugars, organic acids, amino acids and carotenoids. The underlying mechanisms of fruit development and ripening have been extensively studied in tomato [2], but are not well explored in non-climacteric fruits. Citrus is a widely grown fruit crops, which exhibits non-climacteric ripening behaviour. Its fruit contains a juicy pulp made of vesicles within segments [3]. The growth and development of the citrus fruit can be divided into three stages: cell division, an expansion phase involving cell enlargement and water accumulation, and the ripening stage [4]. In the latter stage, carotenoids and other soluble solids are accumulated, chlorophyll is lost, the cell wall is extensively modified, the organic acid content is reduced, and the concentration of a number of volatiles increases.

Citrus fruit provides a convenient vehicle to study gene regulation during non-climacteric fruit development and ripening. In grape (*Vitis vinifera*), another non-climacteric fruit, several comprehensive mRNA expression profiling studies have been presented to describe fruit development and ripening [5-8], while in citrus, an EST sequencing project showed that 20% of the sequences were metallothionein [9]. Application of a citrus cDNA microarray has suggested that unique genetic regulatory networks arise during fruit development [10], while a more global transcriptome analysis was able to identify ethylene-responsive genes in the mandarin fruit [11]. More recently, a comprehensive study of the clementine fruit transcriptome has proposed a mechanism for citrate utilization [12]. However, these transcriptomic studies of fruit development in citrus have mainly been based on microarray analysis, the next generation sequencing technology provides new opportunities for more accurate and powerful deep transcriptome analysis of fruit development.

The ripening of citrus fruit is accompanied by carbohydrate build-up, acid reduction, and carotenoid accumulation. Citrus fruits accumulate most of their sucrose in the juice cells [13]. Glucose is decomposed via glycolysis and the pentose phosphate pathway (OPP). As the major acid present in citrus fruit, citric acid contributes > 90% of the total organic acid content. Citric acid synthesis is thought to take place in the mitochondria via the tricarboxylic acid (TCA) cycle. Although the genes encoding some of the key enzymes (specially, citrate synthase (CS), aconitase [14], and NADP-isocitrate dehydrogenase (NADP-IDH) [15]) have been isolated, their activity cannot completely account for variation in the level of citric acid in citrus fruit [16]. Carotenoids are also important components of the citrus fruit, and their composition and content in sweet orange fruits have been extensively studied [17,18]. Cross-talk between carotenoid, sugar and organic acid metabolism has been documented [19,20]. The presence of sucrose may promote colour break [21], while its deficiency delays lycopene accumulation in tomato [22]. The down-regulation of CS and NAD-dependent IDH results in a decrease in the levels of both organic acids and carotenoids [23].

The fruit of the bud mutant Hong Anliu is characterized by a high sucrose and low citric acid level and its tendency to accumulate lycopene is responsible for the red pigmentation of its pulp [20]. The mutant appears to be highly isogenic with its progenitor wild type variety Anliu, as revealed by a genotypic analysis based on microsatellites and AFLPs. The mutant has therefore provided a platform to study cross-talk between primary (sugar and organic acid synthesis) and secondary (carotenoid synthesis) metabolism enzymes, which are fundamental in the determination of citrus fruit quality. Suppressive subtraction hybridization combined with cDNA microarray analysis has been applied to determine that the differentially expressed genes were mainly enriched in the stage of 170 DAF (days after flowering) in the mutant fruit [24]. At this stage, a total of 582 genes were found to be differentially expressed between the wild type Anliu (hereafter WT) and Hong Anliu (hereafter MT) as revealed by RNA-seq analysis [25]. However, how genes are dynamically and differentially expressed during fruit development and ripening has not yet been determined. Here, the developmental changes of fruit transcriptome of sweet orange were investigated.

## Methods

### Plant material and RNA preparation

WT and MT plants were both cultivated in the same orchard at the Institute of Citrus Research (Guilin, Guangxi Province, China), with the same climatic conditions. Fruit samples were harvested at 120, 150, 190, and 220 DAF (days after flowering) from three different trees in 2009. At each developmental stage, ten representative fruits were sampled from each tree. The pulp was separated from the peel, and the pulp was sliced. The sliced WT pulps samples were combined with one another (similarly for the MT samples), snap-frozen in liquid nitrogen and kept at -80°C until required [24,25]. One aliquot was used to extract RNA isolation, as described previously [26]. The remainder of the powder was used for the determination of sugar and organic acid composition and concentration, and the content of H_2_O_2_.

### RNA-seq and functional assignment

The WT and MT fruit pulp harvested at 120, 150, 190, and 220 DAF was subjected to RNA-seq using an Illumina Genome Analyzer at Beijing Genomics Institute (Shenzhen) in 2009. The abundance of each tag was normalized to one transcript per million (TPM) for between sample comparison purposes. The raw data was filtered to remove low quality sequences including ambiguous nucleotides, adaptor sequences, and below 3 TPM, as described previously [25]. The sequencing data can be accessed at the website: http://www.ncbi.nlm.nih.gov/geo/query/acc.cgi?token=dxqjxoygumyauzm&acc=GSE22505. To link the expressed signatures to known genes from orange, the TIGR unigene dataset (http://compbio.dfci.harvard.edu/tgi/cgi-bin/tgi/gimain.pl?gudb=orange) was used as a reference database. The Z-score method using the p-value as a statistical significance index [27] was applied to identify differentially expressed genes. A cluster analysis was performed according to Eisen et al. [28]; the log_2 _of TPM for each gene was used for the hierarchical clustering analysis. Gene Ontology (GO) categorization was carried out as described previously [25]. The ultra-geometric test was applied to perform GO enrichment analysis. In the significance analysis of the enrichment of a GO item, the p-value represents the probability of satisfying the hypothesis that the designated genes involved in the GO item has not been enriched (statistical significance at P = 0.05).

### Real-time quantitative RT-PCR

The differential expression of a selection of the genes identified as being differentially expressed was validated by applying real-time quantitative RT-PCR (qRT-PCR). The sequences of the primer pairs (designed using Primer Express 3.0 (Applied Biosystems, Foster City, CA, USA)) are listed in additional file 1. All qRT-PCRs were performed using an ABI 7500 Real Time System (Applied Biosystems) using the *actin *gene as the reference [20]. Primers for both the target gene and the reference were diluted in SYBR GREEN PCR Master Mix (Applied Biosystems) and 20 μL of the reaction mix were added to each well. Reactions were performed via an initial incubation at 50°C for 2 min and at 95°C for 10 min, and then cycled at 95°C for 15 s, and 60°C for 60 s for 40 cycles. The resulting data were handled by the instrument on-board software Sequence Detector Version 1.3.1 (Applied Biosystems).

### Analysis of sugar, organic acid and H_2_O_2_

Soluble sugar and organic acid composition and concentrations were determined by gas chromatography (GC) using 3 g of the powdered pulp as described previously [29] with minor modifications. The powder was suspended in chilled 80% methanol and then held in a 75°C water bath for 30 min. After a 2 h ultrasonic extraction and centrifugation at 4000*g *for 10 min, the supernatant was collected and 1 mL internal standard (2.5% w/v phenyl-β-D-glucopyranoside, 2.5% w/v methyl-α-D-glucopyranoside) was added. The solution was made up to 50 mL with 80% methanol, and a 2 mL aliquot was centrifuged at 12000*g *for 15 min. A 0.5 mL aliquot of this final supernatant was vacuum-dried and then re-dissolved in 800 μL 2% w/v hydroxylamine hydrochloride in pyridine at 75°C for 1 h. Then 400 μL hexamethyldisilazane and 200 μL trimethylchlorosilane were added and the sample was held at 75°C for 2 h. A 0.5 μL aliquot was used for GC analysis in an Agilent 6890N device (Santa Clara, CA, USA) equipped with a flame ionization detector. A capillary column (HP-5, 5% phenyl-methylpolysiloxane, 30 m × 25 μm i.d. × 0.1 μm) was employed, with nitrogen as the carrier gas at a flow rate of 45 mL/min, and flow-rates of hydrogen and air set to 40 mL/min and 450 mL/min, respectively. Sugars and organic acids were identified through a comparison of retention times using standard compounds from Sigma (St. Louis, MO, USA). The concentration of H_2_O_2 _was measured using a hydrogen peroxide detection kit supplied by Nanjing Jiancheng Institute of Biological Technology (Nanjing, China). A 0.8 g sample of powdered pulp was suspended in 7.2 ml saline (0.90% w/v of NaCl) and centrifuged for 10 min at 10, 000*g*. The intensity of yellow complex formed by the reaction of molybdate and H_2_O_2_, as measured spectrophotometrically at 405 nm, was used to assess the concentration of H_2_O_2_. Three replicates were conducted for each sample.

## Results

### The fruit transcriptome sampled at four developmental stages

In total, eight cDNA preparations were sequenced from fruit pulp sampled at 120, 150, 190, and 220 DAF from WT and MT. The average number of tags produced for each library was 4.01 million (Table 1). The raw data were submitted and available from the NCBI/GEO repository (accession number GEO: GSE22505; website: http://www.ncbi.nlm.nih.gov/geo/query/acc.cgi?token=dxqjxoygumyauzm&acc=GSE22505). After filtering, the number of robust tags per library ranged from 2.5 to 4.3 million, and the number of distinct tags from 61,000 to 113,000. A saturation analysis (additional file 2) demonstrated that as sequencing depth was increased, the number of new distinct tags decreased, but only until the number of sequences had reached 2.5 million. We concluded therefore that the libraries were all fully saturated and hence large enough for gene expression analysis. The distribution of distinct tag abundance and total tag number exhibited very similar tendencies for all eight libraries (additional file 3). Transcripts which accounted for nearly 60% of the total number were in less than 7% of the categories, and transcripts that accounted for 40% of the categories were less than 5% of the total number, indicating that only a few genes were expressed at a high level.

**Table 1:** Summary of the RNA-seq data collected from MT and WT at each of four selected fruit developmental stages

Category		120DAF		150DAF		190DAF		220DAF	
Total Sequence Collected	WT	2718771		4184144		4417154		4564850	
	MT	3998867		3254246		4275571		4745537	
Low Quality Tags	WT	187214	6.89%	393141	9.40%	356403	8.07%	397623	8.71%
	MT	359939	8.99%	263844	8.11%	401180	9.38%	451906	9.52%
Reliable Tags	WT	2531557	93.11%	3791003	90.60%	4060751	91.93%	4167227	91.29%
	MT	3638928	91.00%	2990402	91.89%	3874391	90.62%	4293631	90.48%
Distinct tags	WT	60841	2.24%	111341	2.66%	95693	2.17%	101845	2.23%
	MT	101301	2.53%	92270	2.84%	104280	2.44%	113006	2.38%

DAF, days after flowering.

### Transcriptome changes during fruit development and ripening

**Figure 1:**
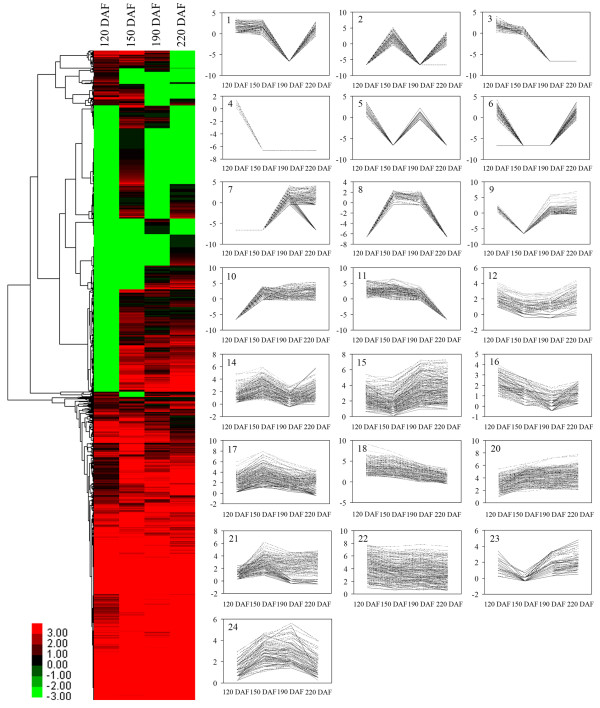
**RNA-seq based transcriptome dynamics of 'Anliu' sweet orange during fruit development and ripening**. The log_2 _of transcripts per million (TPM) for each gene was used for the hierarchical clustering analysis at each of the four selected developmental stages (120, 150, 190 and 220 DAF). The 18,829 genes were classified into 22 regulation patterns (groups 1-12, 14-18, and 20-24). The designation was based on the nomenclature of the gene expression pattern in MT (additional file 6). The gene expression patterns of groups 13 and 19 from MT were absent in WT while two additional patterns, designated as groups 23 and 24, were exclusively present in WT. DAF, days after flowering.

To map tags to known genes, a reference citrus unigene dataset containing 26,826 contigs and 73,607 singletons was used. The procedure identified between 68.1% and 76.2% of the tags (additional file 4), of which 20,155 to 36,173 (31.8% to 33.1%) produced unambiguous identifications (one tag mapping to one gene). The libraries were relatively uniform with respect to mapping efficiency. A total of 18,829 genes were detected in at least one of the four stages in the wild type sweet orange, of which 8,825 genes were expressed in all the four stages. In this study, we solely used the wild type sweet orange as a model to demonstrate the transcriptome changes during fruit development and ripening. Three genes were most highly expressed in wild type, two of which were encoded a stress-response protein (one 22 kDa polypeptide response to low temperature stress) and a heat shock protein, while the function of the third one (F28C11.8) is unknown. Changes in the transcriptome during fruit development and ripening were examined by cluster analysis of gene expression patterns, which arranged the 18,829 genes into 22 groups (Figure 1); the 10,005 (53.1%) genes expressed in three or less of the three stages fell into groups 1 to 11. The largest group (20) comprised the 3,075 (16.3%) genes whose expression increased continuously during fruit development and ripening; this group included the genes encoding sucrose phosphate synthase, cysteine proteinase and a sucrose transporter. The second largest group (10) contained the 2,970 (15.8%) genes which were not expressed at 120 DAF but maintained a stable expression level at other three developmental stages. The 2,618 (13.9%) genes in group 2 were not expressed at 120 and 190 DAF. The cluster analysis also revealed that the abundance of 89.7% of the transcripts detected in the WT pulp varied over the course of fruit development and ripening (Figure 1). Many of the transcripts were single stage-specific (additional file 5). A comparison of expression patterns between WT (Figure 1) and MT (additional file 6) revealed that 20 of the groups were common to both, while 97.5% of the genes expressed in MT showed a similar expression pattern to that in WT (the exceptions belonged to groups 13 and 19).

### Differentially expressed genes during fruit development and ripening

**Figure 2:**
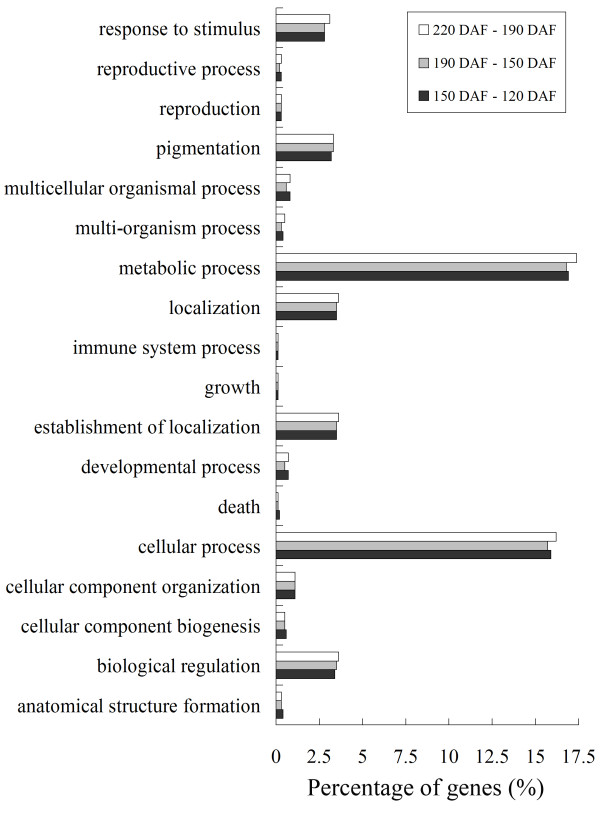
**Functional categorization of genes differentially expressed during fruit development and ripening in 'Anliu' sweet orange based on biological process of Gene Ontology**. The differences between 120 and 150 DAF are indicated by black squares. The differences between 150 and 190 DAF are indicated by light grey squares. The differences between 190 and 220 DAF are indicated by white squares. Percentages are based on the proportion of the number of genes in each set.

Of the 18,829 genes detected in the WT, 9,377, 7,886, and 7,757 were differentially expressed between 120 DAF and 150 DAF, 150 DAF and 190 DAF, and 190 DAF and 220 DAF, respectively. Of these, 36.7% were assigned to one of 18 GO categories (Figure 2). The categories "metabolic process", "cellular process", "establishment of localization", "localization", "biological regulation", "pigmentation", and "response to stimulus" based on biological process captured most of these genes. Several of the differentially expressed genes were associated with cell wall metabolism and softening (Table 2). A gene encoding pectinesterase (*TC14614*) showed a decrease in expression during fruit development and ripening, consistent with the modification of pectin which occurs during ripening. A xyloglucan endotransglycosylase gene (*TC9277*) was up-regulated during fruit development and ripening, while another (*TC25537*) was only detectable at the early stage of fruit development. The abundance of three expansin (*EY741042*, *TC20787*, and *EY738078*) and three alpha-expansin (*EY727139*, *EY743186*, and *EY699153*) transcripts increased during fruit development and ripening. Some genes involved in sucrose metabolism, the TCA cycle and carotenoid biosynthesis were found to be differentially expressed (Table 2). The expression of a sucrose phosphate synthase gene family member (*EY677217*) increased during ripening, and its expression profile correlated well with the rise in total soluble solids. Related genes encoded CS (*EY703799*), aconitase-iron regulated protein 1 (*CB293814*), phytoene synthase (PSY) (*EY722043*) and phytoene desaturase (PDS) (*TC3*). Some of the differentially expressed genes were associated with stress response (Table 2): these included genes encoding a heat shock protein and various metallothioneins. Most of the metallothionein genes were up-regulated in the later stages of ripening. Three cysteine protease genes (*EY718215*, *TC22740*, and *EY707198*), were also highly expressed at the late stage of fruit development and ripening. Also, the expression of a valencene synthase gene (*TC363*) increased during fruit development and ripening.

**Table 2:** List of 10 genes involved in major processes associated with fruit development and ripening based on Gene Ontology categorization

Go term	Gene identifier	Gene discription	150vs120 Fold change	190vs150 Fold change	220vs190 Fold change
carotenoid	TC14030	Zeta-carotene desaturase	4.94	-	2.19
metabolic	TC15628	Zeta-carotene desaturase	6.59/0	-	2.93
process	TC19375	carotenoid isomerase	3.33	0.19	-
	TC3	Phytoene desaturase	6.18	-	2.19
	TC4815	Epsilon lycopene cyclase	3.1	-	-
	TC5834	Capsanthin/capsorubin synthase	0.35	8.39	0.55
	CN187831	Zeta-carotene desaturase	-	-	1.2/0
	EY722043	Phytoene synthase	-	1.36	-
	TC26011	Zeta-carotene desaturase	-	0/3.43	1.2/0
	TC5	Lycopene beta-cyclase	-	2.88	-
cell wall	EY676350	Reversibly glycosylated protein	0.13	9.53	1.81
organization	EY727139	Alpha-expansin 3	0/1.19	-	-
	TC4398	Pectinesterase-3 precursor	0.57	1.43	0.31
	EY700757	Pectinesterase-1 precursor	-	-	0.26
	EY743186	Alpha-expansin precursor	-	1.48/0	-
	TC14614	Pectinesterase PPE8B precursor	-	0.09	0/0.99
	TC25537	Xyloglucan endotransglucosylase	-	0/1.58	-
	EY738078	Expansin	-	5.17/0	-
	EY679620	Cellulose synthase	0.3	0.33	0.2
	EY679934	Cellulose synthase	0.51	0.23	-
sucrose	TC11046	Sucrose synthase	0.32	0.39	0.41
metabolic	EY743126	Sucrose-phosphate synthase 1	7.65/0	-	1.77
process	TC14378	Sucrose-phosphate synthase 1	3.28	-	2.53
	TC20096	Sucrose synthase	1.85/0	0/1.85	1.44/0
	EY719381	Sucrose-phosphate synthase 1	-	-	0.37
	CX044648	Sucrose synthase	-	0.1875	-
	EY667747	Sucrose-phosphate synthase	-	-	-
	EY677217	Sucrose-phosphate synthase	-	-	8.43
oxygen and	EY680864	Superoxide dismutase	5.28/0	-	0.25
reactive	EY741543	Superoxide dismutase [Cu-Zn]	1.78	-	1.69
oxygen	TC12069	Cu/Zn superoxide dismutase	32.18/0	0.62	-
species	TC14743	Cu/Zn-superoxide dismutase copper chaperone precursor	2.71	-	-
	TC2154	Superoxide dismutase [Cu-Zn]	2.67	0.36	3.72
	TC22348	Superoxide dismutase	4.22	-	-
	TC23992	Superoxide dismutase	11.34/0	0.61	2.47
	CN189455	Superoxide dismutase [Mn], mitochondrial precursor	3.69/0	2.07	-
	TC4680	Superoxide dismutase	3.55	2.87	1.48
	TC19068	Cu/Zn-superoxide dismutase copper chaperone precursor	-	-	-
Response	CF836240	Peroxidase precursor	0.31	-	0/3.69
to stress	CK701553	Heat shock protein 70	0.19	-	0.26
	EY676086	Serine/threonine-protein phosphatase PP1	2.37/0	-	-
	EY735114	Plastid enolase	4.68	-	2
	TC18825	2-oxoacid dehydrogenase family protein	3.97	0.54	2.81
	TC21917	Methionine synthase	2.11/0	-	-
	TC3575	22 kDa polypeptide	1.12	-	0.92
	EY730377	Group 5 late embryogenesis abundant protein	-	-	2.03
	TC20176	Osmotin-like	-	-	0/0.99
	TC8117	Thaumatin-like protein	-	20.91	3.63

For each gene, the number given at each stage indicates that the level of expression differed significantly compared with the level at the former stage; -, no significant difference; 0, zero detectable expression, for example, 6.59/0 indicates that the expression was not detected at the earlier sampling stage, but showed a transcript level of 6.59 at the current one.

### Developmental difference between fruit transcriptome of MT and WT

**Figure 3:**
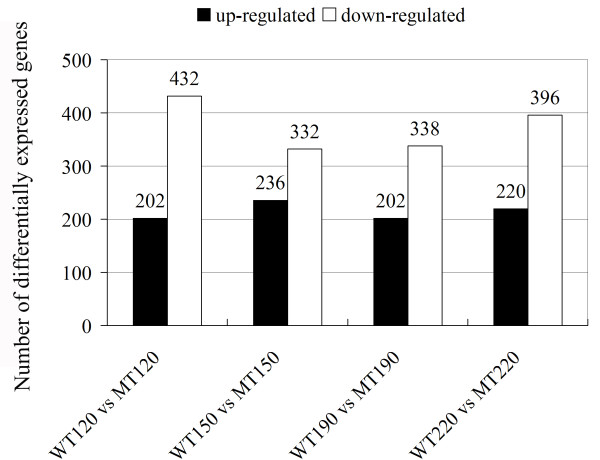
**Distribution of genes differentially expressed between 'Anliu' sweet orange and its red flesh mutant at each of four selected stages of fruit development and ripening**. The number of up-regulated genes was less than that of down-regulated ones at all the four stages. WT120 vs MT120 indicates a comparison between WT and MT at 120 DAF.

The comparison between the transcriptomes of WT and MT identified 6,540 genes showing a two-fold or greater difference in expression level at 120 DAF, 3,529 at 150 DAF, 4,601 at 190 DAF, and 3,289 at 220 DAF. Application of the Z-score method [27] suggested that the expression of 634, 568, 540, and 616 of these was significantly different at p < 0.05 and |log_2_Ratio| ≥ 1 in the four developmental stages, respectively (additional file 7). The top ten differentially expressed genes in each of the four developmental stages have been listed in additional file 8. Many encode stress-related products, such as cysteine protease Cp5 and metallothionein-like protein. At all the four developmental stages the number of up-regulated genes was less than that of down-regulated genes (Figure 3).

When the expression profiles of the genes differentially expressed between MT and WT were subjected to a cluster analysis (additional file 9), over one half (492/883) turned out to be up-regulated in MT at all the developmental stages except 150 DAF. Some examples of this large cluster included genes encoding Cu/Zn superoxide dismutase (*TC12069*), ascorbate peroxidase (*EY685405*, *TC22775 *and *TC14669*), MYB1 (*EY649968*) and plastidic glucose 6-phosphate/phosphate translocator (*EY722703*). Only five genes were detected as differentially expressed at all four stages; one encoded a cysteine protease (Cp5) (*TC5370*), which had been reported exhibiting the acid-activatable cysteine protease forms [30], three had no assigned functions, and one shared no homology with any entry in. The five genes could be classified into two groups (two genes in one group, three genes in the other), with their expression patterns of being opposite to each other but with both groups having a turning point at 150 DAF (additional file 10).

The GO categories of the set of differentially expressed genes (additional file 11) revealed that most encoded products associated with "protein binding", "hydrolase activity", "transferase activity" and "transporter activity". At the 150 DAF stage, the most common categories were "signal transducer activity" and "electron carrier activity". GO enrichment analysis was also carried out to identify which key processes were altered in MT (Table 3). The genes encoding phytoene synthase (*EY722043*) and ζ-carotene desaturase (ZDS) (*TC15628*) involved in carotenoid metabolic process were enriched at 150 DAF in MT compared to WT, and those encoding capsanthin/capsorubin synthase (CCS) (*TC5834 *and *TC9367*) were also enriched at 150 DAF in MT.

**Table 3:** Gene ontology enrichment analysis for the genes differentially expressed between WT and MT during fruit development and ripening

Category	Library pair	Enriched GO terms	Cluster frequency	P-value	Genes in group
Molecular function	WT150vsMT150	capsanthin/capsorubin synthase activity	1.2%	0.003	TC5834, TC9367
		sulfotransferase activity	1.5%	0.010	TC5490, DN620599
	WT190vsMT190	transferase activity, transferring glycosyl groups	9.3%	0.006	EY691346, TC18663, TC10188, TC8764, TC320, TC9277, TC11658, EY679857, TC24546, TC11943, TC8402, CK939135, TC15309, TC22218, EY661193
		protein N-terminus binding	1.2%	0.031	TC2476, TC10188
	WT220vsMT220	fructokinase activity	1.5%	0.019	TC9238, EY702245, TC17456
		sulfotransferase activity	1.0%	0.030	TC5490, DN620599
Biological process	WT120vsMT120	cell wall organization	5.1%	0.021	TC4981, CK933828, DN618740, EY725863, TC18738, TC3660, TC12562, EY746957, TC14614
		protein import into nucleus, docking	1.7%	0.034	EY701416, EY733548, DY305879
	WT150vsMT150	carotenoid metabolic process	3.3%	0.043	EY722043, TC15628, TC5834, TC9367

### Verification of differentially expressed genes during fruit development and ripening

**Figure 4:**
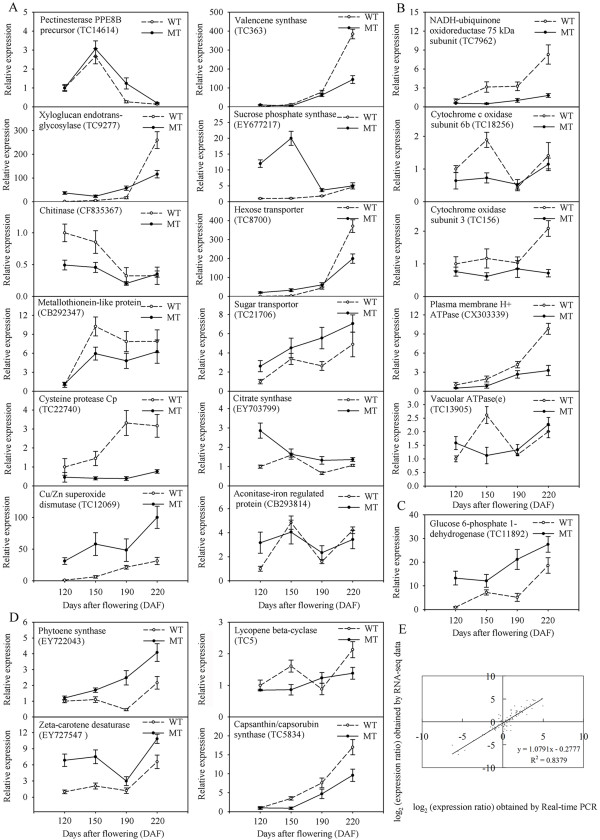
**qRT-PCR validation of differential expression**. The figure shows transcript levels of 22 genes, of which 12 probably associated with fruit development and ripening (A) and 10 with oxidative phosphorylation (B), the OPP pathway (C) or carotenoid biosynthesis (D) in MT (solid line) and WT (broken line). The *y*-axis records the relative gene expression levels analyzed by qRT-PCR. Bars represent the standard error (n = 3). (E) A comparison between the gene expression ratios obtained from RNA-seq data and qRT-PCR. The RNA-seq log_2 _of expression ratio (*y*-axis) have been plotted against developmental stages (*x*-axis).

RNA sampled from fruit of MT and WT at the four selected stages of fruit development and ripening provided the template for qRT-PCR validation of the sequence-based transcription profiles for 22 of the differentially expressed genes (Figure 4). Linear regression [(RNA-seq value) = *a*(RT-PCR value) + *b*] analysis showed an overall correlation coefficient of 0.8379, indicating a good correlation between transcript abundance assayed by real-time PCR and the transcription profile revealed by RNA-seq data (Figure 4E).

### Changes in fruit soluble sugars, organic acids, carotenoids and H_2_O_2 _content

**Figure 5:**
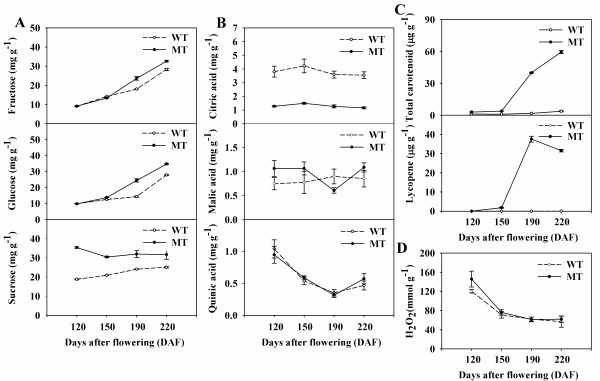
**Dynamics of soluble sugar, organic acid, total carotenoid, lycopene and H_2_O_2 _accumulation during fruit development and ripening **. The concentrations of soluble sugars (A) and organic acids (B) were determined by GC. The total carotenoid and lycopene (C) contents have been published previously [20]. The H_2_O_2 _content (D) was determined by spectrophotometer at 450 nm. 'Anliu' sweet orange (WT, broken line) and its red flesh mutant (MT, solid line) fruits at each of four selected developmental stages were used in the analysis. Bars represent the standard error (n = 3). DAF, days after flowering.

Since the expression of a number of genes implicated in carbohydrate metabolism and mitochondria-related citric acid metabolism varied greatly during fruit development and ripening, an attempt was made to monitor the dynamics of pulp soluble sugar and organic acid content. The content of soluble sugars increased markedly during the late stages of fruit development and ripening in both WT and MT (Figure 5A). The concentrations of glucose and fructose remained rather constant up to 150 DAF, and thereafter tended to be higher in MT than in WT. The concentration of sucrose was higher in MT than in WT throughout fruit development and ripening. The citric acid content fluctuated, but was lower in MT than in WT at all developmental stages (Figure 5B). The concentration of quinic acid decreased substantially over the course of fruit development and ripening, but did not differ between WT and MT. Carotenoids and lycopene both accumulated over time in MT, but remained at a low level in WT (Figure 5C). Finally, H_2_O_2 _content fell as the fruit developed and ripened, but was higher in MT than in WT at 120 DAF (Figure 5D).

## Discussion

### Transcriptome dynamics during fruit development and ripening

Fruit ripening is a highly coordinated, genetically programmed and irreversible process which involves a series of physiological, biochemical, and organoleptic changes allowing for the development of an edible ripe fruit [31]. Fruit ripening in citrus is accompanied by carbohydrate build-up, acid reduction, carotenoid accumulation and chlorophyll degradation [32]. The bud mutant MT produces fruit with high sucrose and lycopene, but low citric acid content [20]. Here, transcriptome changes over the course of fruit development and ripening in MT and WT were monitored and annotated. We must point out that we collected samples from three different trees and pooled for RNA-seq analysis for each developmental stage. We did not include biological replicates considering that we used a pair of genotypes (WT and MT) and the data from WT and MT could corroborate to each other to some extent. We sequenced each pool once technically since the next generation sequencing data are highly replicable with relatively little technical variation [33]. We also used real-time qRT-PCR to verify the transcription profile revealed by RNA-seq data (Figure 4), with an overall correlation coefficient of 0.8379 which indicated the RNA-seq data was reliable. The RNA-seq approach detected a similar number of genes (18,829 genes in WT and 19,440 genes in MT) in both genotypes, with no apparent difference between the global number of genes expressed in the two genotypes at any of the four stages of fruit development and ripening sampled (additional file 4). The WT and MT patterns of gene expression were also largely alike (20 of the 24 expression patterns were similar). Thus, we believed that overall picture of the transcriptome captured by RNA-seq is robust.

An overwhelming proportion of the genes identified (84.8% in MT and 89.7% in WT) varied in their level of expression over the course of fruit development and ripening, reflecting the occurrence of a massive genetic re-programming. A large number of these genes were expressed in a stage-specific manner, which implicates their involvement in physiological processes which take place only at a specific developmental stage(s). A major group of the differentially expressed genes was involved in cell wall modification, which is not surprising since the major textural changes associated with the softening of fruit are due to enzyme-mediated alterations in the structure and composition of the cell wall [34], especially the cell walls of juice sacs in citrus. Changes in the activity of several cell wall-related genes were known to result in the abnormal development of juice sac granulation [35,36], while modifications in cell wall structure or in the components of the membranes of the segments and juice sacs during fruit development and ripening clearly influenced the formation of the fruit pulp melting characteristic [37]. One of the cell wall-related genes revealed here encoded a pectinesterase, an enzyme which modifies the assembly and disassembly of pectin, a common component of the primary cell wall. In tomato, the gene for pectinesterase was highly expressed prior to ripening, and was down-regulated by ethylene as ripening begins [38]. Here, the expression of the gene encoding the pectinesterase PPE8B precursor decreased as the WT fruit matured (Figure 4A), while in the blood orange, the expression of a pectinesterase gene has been shown to increase during fruit development and ripening [39]. It might be due to the different members identified in the two studies. The hemicellulose xyloglucan is a common component of the cell wall, and is hydrolysed and transglycosylated by xyloglucan endotransglycosylase in growing tissues and ripening fruits [40]. Here, a gene encoding this enzyme was up-regulated during fruit development and ripening (Figure 4A), indicating its probable role in cell wall degradation during ripening. Both the early enlargement of the citrus fruit driven by cell expansion and the later ripening process require the presence of expansins to loosen the cell walls [41], and several genes encoding expansins were detected in the present study and their expression was higher at the later stages of fruit development and ripening.

The accumulation of carbohydrates represents one of the most obvious changes which occur during citrus fruit development and ripening. The perceived changes in expression of genes involved in carbohydrate metabolism here were consistent with the findings of other transcriptomic analyses in citrus [12]. The type of sugar deposited to a high level in the cell vacuole in citrus is predominantly sucrose, unlike in grape, where it is glucose and fructose [6]. Reflecting this difference, the expression profile of the gene encoding sucrose synthase in citrus [12] was rather different from that in the grape [6]. Nevertheless, in both species, sugar is important for the regulation of colour development, and perhaps also for other ripening processes [42]. It was notable that the gene encoding a sucrose phosphate synthase was induced during fruit development and ripening, perhaps because the activity of this enzyme may be required for the re-synthesis of sucrose to allow its further transport to the vacuole [43]. Of some interest also was the behaviour of a gene encoding valencene synthase, an enzyme the activity of which was known to be induced by ethylene, and which was part of the natural ripening process in citrus [11]. Valencene is an important component of the aroma of the ripe sweet orange fruit. Both in the present experiments (Figure 4A) and elsewhere [44], valencene synthase transcript accumulated in the ripening fruit.

### Molecular processes involved in the formation of the fruit traits of red flesh sweet orange

Suppressive subtraction hybridization, in combination with cDNA microarray analysis, has identified a set of 267 genes which were differentially expressed between MT and WT [24]. RNA-seq technology is more capable of identifying a nearly complete inventory of transcripts and by this method a total of 582 genes were found to be differentially expressed between WT and MT at the stage of 170 DAF [25]. In the current study, we tested on four developmental stages of WT and MT and extended our understanding of the global and dynamic changes during fruit development and ripening in MT and WT. Almost all of the members of the 267 gene set revealed by SSH strategy were also identified in the present study, although some discrepant expression patterns were apparent between this new data set and previously assembled microarray-based set. For example, the abundance of the transcript encoding cysteine protease, which appeared to differ between MT and WT in both studies, was documented by the microarray analysis as being lower at all developmental stages, whereas it appeared to be higher at 150 DAF in MT in the present study (additional file 10).

The major biological processes occurring in the mitochondria (TCA cycle, coupling electron transfer, and oxidative phosphorylation) were remarkably altered in MT. The intermediates of the TCA cycle can be channelled into the syntheses of fats, terpenoids, porphyrins, nucleotides, and amino acids. In MT, the level of citric acid, the major organic acid present in citrus fruit, was consistently around 25% that present in WT fruit (Figure 5). However, no major difference was detected in the expression of the genes encoding CS and aconitase-iron regulated protein (Figure 4A), two predominant enzymes involved in the TCA cycle. Five differentially expressed genes, all associated with mitochondria-related processes (coupling electron transfer and oxidative phosphorylation) were down-regulated in MT compared with WT (Figure 4B). These included the genes encoding a NADH-ubiquinone oxidoreductase 75kDa subunit (*TC7962*), cytochrome C oxidase (*TC18256*), and cytochrome oxidase subunit 3 (*TC156*), suggesting that MT mitochrondria were capable of less efficient electron transport than that WT ones. If, as a result, flux through the TCA cycle is decreased, the accumulation of citric acid is likely to be compromised. In addition, PDS and ZDS catalyzing desaturation of phytoene to lycopene involve net electron transfer [45]. In tomato, a NAD(P)H dehydrogenase complex which participating in electron transfer was involved in carotenoid biosynthetic pathway [46], suggesting the possible exist of cross-talk between electron transfer and carotenoid accumulation in sweet orange.

In the plant cell, the mitochondrial electron transport chain is a major site of reactive oxygen species (ROS) production [47]. Here, the concentration of one of the primary ROS molecules (H_2_O_2_) was higher in MT than that in WT at 120 DAF (Figure 5D). The delicate balance between antioxidant defence and ROS production can be disrupted by either compromised antioxidant defence or the inhibition of electron flow [48]. Here, the primary anti-oxidant enzymes (SOD, APX and GR) were more active in MT than in WT pulp, suggesting that the level of oxidative stress may be greater in MT than in WT [49]. The expression of a large number of stress-related genes was also substantially different in MT and WT (additional file 8). Functioning as an important ROS scavenging pathway, the ascorbate-glutathione cycle requires the cofactor NADPH, which is provided by the OPP pathway [50]. This pathway is also a major source of NADPH for many biosynthetic processes, including carotenoid biosynthesis [51], in non-photosynthetic organs such as the fruit. The gene encoding glucose 6-phosphate dehydrogenase, which is considered as the first and rate-limiting enzyme of the OPP pathway in all cells [52], was up-regulated in MT. A statistical analysis of the qRT-PCR result confirmed that the level of transcription of this gene was significantly higher in MT than in WT, especially at 120 DAF (Figure 4C). Carotenoid biosynthesis, another effective anti-oxidative process [53], was also higher in MT than in WT. Lycopene is the most potent antioxidant among the carotenoids [54]. The expression level of several carotenoid biosynthesis genes, encoding namely PSY, ZDS, lycopene β-cyclase (LCYb) and CCS, was greatly changed in MT. PSY is generally accepted to be a rate-limiting enzyme in carotenoid biosynthesis pathway. The *CCS *product is an enzyme which is mechanistically similar to LCYb, and the low transcript level of *CCS *may well be responsible for the accumulation of lycopene in red grapefruits (*Citrus paradisi*) [19]. The qRT-PCR analysis confirmed that both upstream genes (*PSY *and *ZDS*) were up-regulated and both downstream ones (*LCYb *and *CCS*) down-regulated in MT (Figure 4D), consistent with the mechanism regulating lycopene accumulation in tomato [55].

## Conclusion

The present study has provided a dynamic view of the transcriptome during fruit development and ripening of a sweet orange red-fleshed mutant and its progenitor wild type. Cell wall biosynthesis, carbohydrate metabolism, the TCA cycle, and carotenoid biosynthesis were all differentially regulated during fruit development and ripening. These differentially regulated processes may well be important for the formation of the pleiotropic fruit trait of Hong Anliu sweet orange.

## Authors' contributions

KQY, QX and XLD were responsible for generating the RNA-seq data and for the interpretation of the data. KQY carried out qRT-PCR experiments and measured sugar, organic acid and H_2_O_2 _content, and drafted the manuscript. QX conceived the study, participated in its design and helped to draft the manuscript. FG and YDD participated in the statistical analyses. XXD proposed and supervised the research. All authors read and approved the final manuscript.

## Supplementary Material

Additional file 1 **The primer sequence information**. This file listed the primers sequences used for real-time quantitative RT-PCR validation of RNA-seq data. Click here for file

Additional file 2 **The saturation evaluations of the eight libraries in this study**. This file contained the information of the saturation evaluations of the RNA-seq tags in the eight libraries (MT and WT at four selected fruit developmental stages) against sequencing depth. The results revealed that with the increase of total sequence number (sequencing depth), the number of genes identified increased, but the number stabilized once the number of sequences reached 2.5 million, indicating enough information has been included in the RNA-seq data. Click here for file

Additional file 3 **Distribution of total tags number (A) and distinct tags number (B) in MT and WT at 120, 150, 190, and 220 DAF**. This file showed the distribution of the number of total tags and distinct tags obtained in MT and WT at different developmental stages. Click here for file

Additional file 4 **Summary of tags mapped against a reference set of sweet orange unigenes**. This file contained the summary result of tags mapping against a reference set of sweet orange unigenes. Click here for file

Additional file 5 **Number of stage-specific genes expressed in MT and WT**. This file contained the summary result of stage-specific genes number in MT and WT. Click here for file

Additional file 6 **Transcriptome dynamics in MT during fruit development and ripening**. This file contained the result of the hierarchical cluster analysis of genes expression profiles in MT. The log_2 _of transcripts per million (TPM) for each gene was used for the hierarchical clustering analysis at four developmental stages (120, 150, 190 and 220 DAF). In all, 19,440 genes were classified into 22 regulatory patterns, designated groups 1-22. Click here for file

Additional file 7 **List of differentially expressed genes between MT and WT**. The table contained information of the differentially expressed genes with expression difference > 2, and genes differentially expressed at 0.05 significance level at each of the four fruit developmental stages. Click here for file

Additional file 8 **The ten most differentially expressed genes between MT and WT at each of the four selected fruit developmental stages**. This file listed the ten most differentially expressed genes between MT and WT at different developmental stages, with their expression ratios between MT and WT, also containing simple annotation information. Click here for file

Additional file 9 **Dynamics patterns of gene expression of a set of genes differentially expressed between MT and WT at each of the four selected fruit developmental stages**. This file contained the result of the hierarchical cluster analysis of expression profiles of differentially expressed genes between MT and WT at different developmental stages. The log_2 _of the ratio between the MT and the WT TPM for each gene was used to perform the cluster analysis. Click here for file

Additional file 10 **The five genes differentially expressed at all four selected developmental stages**. This file contained the pattern of genes which were differentially expressed at all selected stages. At each stage (120, 150, 190 and 220 DAF), the log_2 _of the ratio between the MT and the WT TPM for each gene is represented. Click here for file

Additional file 11 **Functional categorization of genes differentially expressed between WT and MT**. This file showed the distribution of GO categories of differentially expressed genes between WT and MT at the four selected stages of fruit development and ripening. The categorization was based on molecular activity of Gene Ontology items. Percentages are based on the proportion of the number of genes in each set. Click here for file
