# Supplementary material for: Transcriptome changes during fruit development and ripening of sweet orange (Citrus sinensis)
Source: BMC Genomics. 2012 Jan 10;13:10. doi: 10.1186/1471-2164-13-10 (PMC3267696; doi:10.1186/1471-2164-13-10)
Supplement: Additional file 1 — The primer sequence information. This file listed the primers sequences used for real-time quantitative RT-PCR validation of RNA-seq data. [file 1471-2164-13-10-S1.DOC]

**Additional file 1** **Primer sequences used for qRT-PCR validation of RNA-seq data.** The results of the first 23 genes were used for linear regression analysis based on qRT-PCR and RNA-seq analysis.

| Gene | Forward | Reverse |
| --- | --- | --- |
| TC18748 | AGGCCATTCCAGTCAGAGTCA | AATTAAGCTCCGAAGCATCCAA |
| TC599 | GCTCTCCGCGAGCTCCTT | GAGGATGACACCACTGAGGTACTG |
| EY714855 | CAACGACTATGGCTAACACTCTCAA | TGCCATGGATCCTGTTCTTG |
| TC21706 | GGCCGTCGTAGGGCTTTT | TGCACTTATTGAAGCACCAATGA |
| CB292347 | AAAGAGCCCTCTATCCCTTGATTT | GCAGGTGTCCGACATGTTGA |
| TC14251 | GGCCAACAAATATGGACCTTTG | ACGCGACGACTAGGGACATT |
| TC5370 | ATGGGTTATTAACAACGGTGGAA | ACCATCGACACCAGTGTAAGGA |
| TC6198 | TGGTGAACCTCGCAGCTTTC | AAGTTCTCACCATGGCCAGTTT |
| TC14003 | TCCATTGAGGTTGGAACAGTTG | AAACATGCTGACTGTGATGCA |
| TC7121 | GGCACCGGTTCCTGAAGA | CACGACGAGCTTATCCAAAAGC |
| TC12069 | CGAGATCACGTAGCACAATGGT | TTCCAACACGACCCACAACTC |
| TC5834 | CCTTGGCTCAACCAGGATGA | TTGGCCACAACCCATTCC |
| TC5 | CTATATGGTGGCAAGGACTTTAGCT | AGGCTTCGAACGATTGCATT |
| EY727547 | GCTAGTTGGAGTGCCTGTTGTG | TGCAACTCAGTAACCCAACCAT |
| CX047553 | TGCCGTGGTCGGAATCA | ACGCGGGAGACGATGAAC |
| TC7962 | ACGTTGCCTGCAGTTCCAA | TCAGAAAGTGCCCGAATTATCTTC |
| TC18256 | GAACGAGCAGAGGGAAAATGG | GCCACCCGAGTGCTGATG |
| TC19018 | CGAGGGCAAGTGCTCCATA | CTACACTGGATTCTCATGGACTTGA |
| TC156 | GGTGCAACACTTCTCAGTTTGG | AGAACATCGCGCCACCAT |
| CX303339 | TTCGGAATCCGCTACATCCT | TGGTAGTGAAGGCAGTCTTGTTCT |
| TC13905 | GCCACAGTCTCCTGCTGGAT | GGCTTCATTTGCGCCAGATA |
| EY722043 | CCGCGGTCTTCCAGTTATATG | TCGACCGGAATTTGTTACTGATT |
| TC11892 | GCATAAACCCCTTATTGAAATTGG | TCTCAGCACATCGTTTTTTAGCA |
| CB293814 | TGCTTGTTGTTCCTCCTGGTT | CCCGTCCAAGGTATTCCAAGT |
| TC14614 | CACAGCAGGCCCAGAGAAA | CCGAGAGGTCGGAGTCAGATC |
| TC9277 | CAGATGGGTTCGCCAGAAAT | GGGAAGCGGGCACGAT |
| CF835367 | GGTCCCCGTTTGGACAAAGT | ATGATGCCTTTGTTGGCAACT |
| TC22740 | AGCATATCCCTACACCGGAAAA | GGACTTGGACGCCAACATTT |
| TC363 | GCTTGCGGAACAGATAAATCATG | CGCCTCTAATCTTGGTAGGGTTT |
| EY677217 | GTCCGGTGCCTTGAATGTG | CGAACTTATTCCGCCCCAAT |
| TC8700 | TTCTTGCTCCCCGAGACAAA | CCAATGCTGCTTCCACACTCT |
| EY703799 | GCACAGTGAGATTGCAAGCAA | CCACCAGAAGGAGTTGAATTTGA |
