# Supplementary material for: Transcriptome changes during fruit development and ripening of sweet orange (Citrus sinensis)
Source: BMC Genomics. 2012 Jan 10;13:10. doi: 10.1186/1471-2164-13-10 (PMC3267696; doi:10.1186/1471-2164-13-10)

**Additional file 2 Saturation evaluation of the RNA-seq tags in the eight libraries (MT and WT at four selected fruit developmental stages) against sequencing depth.** As the sequencing depth was increased, the number of genes identified increased, but the number stabilized once the number of sequences reached 2.5 million. MT: Hong Anliu; WT: Anliu wild type. MT 120: sampled from MT at 120 DAF (days after flowering).


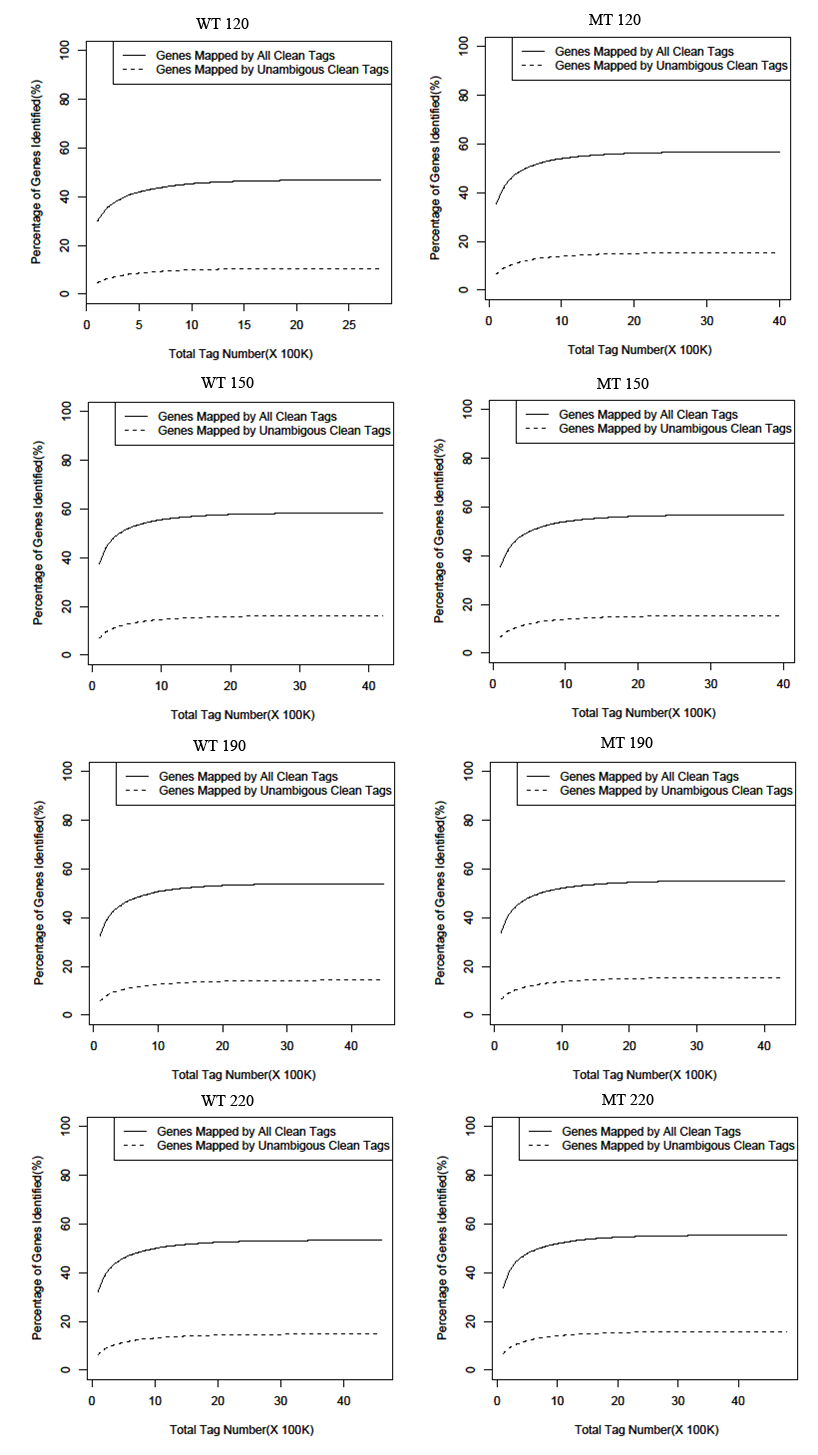

Supplement: Additional file 2 — The saturation evaluations of the eight libraries in this study. This file contained the information of the saturation evaluations of the RNA-seq tags in the eight libraries (MT and WT at four selected fruit developmental stages) against sequencing depth. The results revealed that with the increase of total sequence number (sequencing depth), the number of genes identified increased, but the number stabilized once the number of sequences reached 2.5 million, indicating enough information has been included in the RNA-seq data. [file 1471-2164-13-10-S2.DOC]
