# Supplementary material for: Transcriptome changes during fruit development and ripening of sweet orange (Citrus sinensis)
Source: BMC Genomics. 2012 Jan 10;13:10. doi: 10.1186/1471-2164-13-10 (PMC3267696; doi:10.1186/1471-2164-13-10)
Supplement: Additional file 3 — Distribution of total tags number (A) and distinct tags number (B) in MT and WT at 120, 150, 190, and 220 DAF. This file showed the distribution of the number of total tags and distinct tags obtained in MT and WT at different developmental stages. [file 1471-2164-13-10-S3.DOC]

**Additional file 3 Distribution of total tags number (A) and distinct tags number (B) in MT and WT at 120, 150, 190, and 220 DAF.** DAF, days after flowering.

**
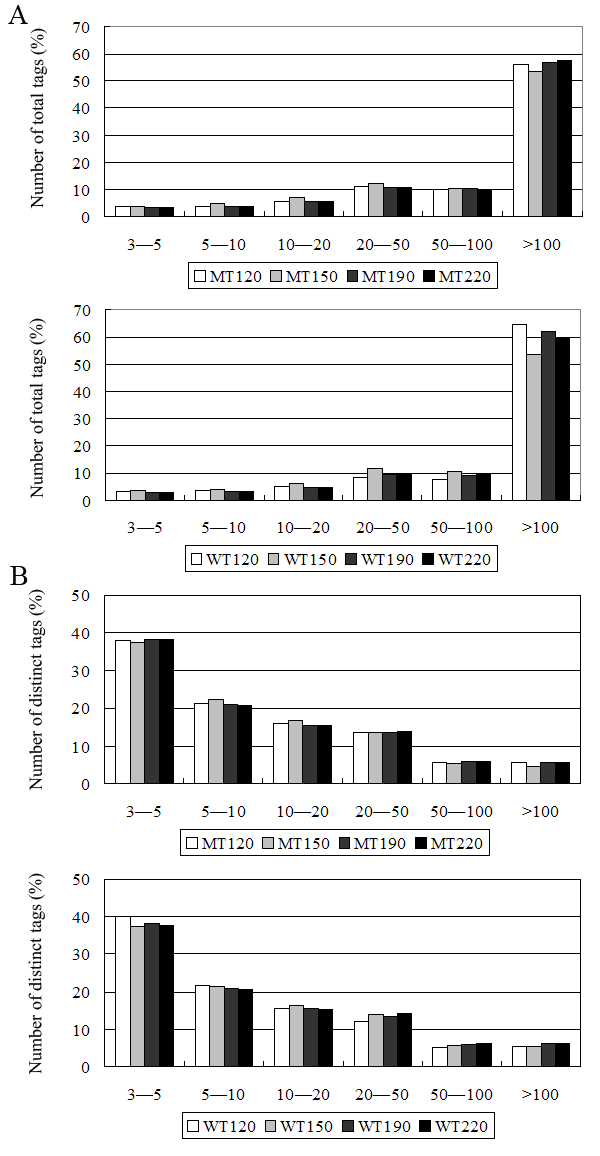
**
