# Supplementary material for: Transcriptome changes during fruit development and ripening of sweet orange (Citrus sinensis)
Source: BMC Genomics. 2012 Jan 10;13:10. doi: 10.1186/1471-2164-13-10 (PMC3267696; doi:10.1186/1471-2164-13-10)
Supplement: Additional file 4 — Summary of tags mapped against a reference set of sweet orange unigenes. This file contained the summary result of tags mapping against a reference set of sweet orange unigenes. [file 1471-2164-13-10-S4.DOC]

**Additional file 4 Summary of tags mapped against a reference set of sweet orange unigenes.**

| **Category** |  | **120 DAF** |  | **150 DAF** |  | **190 DAF** |  | **220 DAF** |  |
| --- | --- | --- | --- | --- | --- | --- | --- | --- | --- |
| Distinct Tags | WT | 60841 |  | 111341 |  | 95693 |  | 101845 |  |
|  | MT | 101301 |  | 92270 |  | 104280 |  | 113006 |  |
| All Tags Mapping to Gene | WT | 46328 | 76.15% | 76424 | 68.64% | 67748 | 70.80% | 69720 | 68.46% |
|  | MT | 70657 | 69.75% | 64493 | 69.90% | 70967 | 68.05% | 75732 | 67.02% |
| Unambiguous Tags Mapping to Gene | WT | 20155 | 33.13% | 36173 | 32.49% | 30998 | 32.39% | 32929 | 32.33% |
|  | MT | 33079 | 32.65% | 30520 | 33.08% | 33442 | 32.07% | 35955 | 31.82% |
| Unknown Tags | WT | 14501 | 23.83% | 34897 | 31.34% | 27935 | 29.19% | 32114 | 31.53% |
|  | MT | 30634 | 30.24% | 27772 | 30.10% | 33281 | 31.92% | 37208 | 32.93% |
| Unambiguous Tag-mapped Genes | WT | 11448 | 11.40% | 17503 | 17.42% | 15526 | 15.46% | 16082 | 16.01% |
|  | MT | 16666 | 16.59% | 15618 | 15.55% | 16625 | 16.55% | 17203 | 17.12% |

Data from four developmental stages of the mutant (MT) and wild type (WT) sweet oranges were used. DAF, days after flowering.
