# Supplementary material for: Transcriptome changes during fruit development and ripening of sweet orange (Citrus sinensis)
Source: BMC Genomics. 2012 Jan 10;13:10. doi: 10.1186/1471-2164-13-10 (PMC3267696; doi:10.1186/1471-2164-13-10)
Supplement: Additional file 5 — Number of stage-specific genes expressed in MT and WT. This file contained the summary result of stage-specific genes number in MT and WT. [file 1471-2164-13-10-S5.DOC]

**Additional file 5 Number of stage-specific genes expressed in MT and WT.**

| **Fruit development stage**  **(Days after flowering)** | **MT**  **(number of** **specifically expressed genes)** | **WT**  **(number of specifically expressed genes)** |
| --- | --- | --- |
| 120 | 543 | 199 |
| 150 | 525 | 1190 |
| 190 | 385 | 289 |
| 220 | 610 | 515 |
| 120/150 | 256 | 246 |
| 120/190 | 135 | 48 |
| 120/220 | 197 | 25 |
| 150/190 | 216 | 394 |
| 150/220 | 210 | 434 |
| 190/220 | 389 | 233 |
| 120/150/190 | 224 | 243 |
| 120/150/220 | 229 | 78 |
| 120/190/220 | 308 | 37 |
| 150/190/220 | 428 | 832 |
| 120/150/190/220 | 2031 | 1021 |
