# Supplementary material for: Transcriptome changes during fruit development and ripening of sweet orange (Citrus sinensis)
Source: BMC Genomics. 2012 Jan 10;13:10. doi: 10.1186/1471-2164-13-10 (PMC3267696; doi:10.1186/1471-2164-13-10)
Supplement: Additional file 6 — Transcriptome dynamics in MT during fruit development and ripening. This file contained the result of the hierarchical cluster analysis of genes expression profiles in MT. The log2 of transcripts per million (TPM) for each gene was used for the hierarchical clustering analysis at four developmental stages (120, 150, 190 and 220 DAF). In all, 19,440 genes were classified into 22 regulatory patterns, designated groups 1-22. [file 1471-2164-13-10-S6.DOC]

**Additional file 6 Transcriptome dynamics in MT during fruit development and ripening.** The log_2_ of transcripts per million (TPM) for each gene was used for the hierarchical clustering analysis at four developmental stages (120, 150, 190 and 220 DAF). In all, 19,440 genes were classified into 22 regulatory patterns, designated groups 1-22.

**
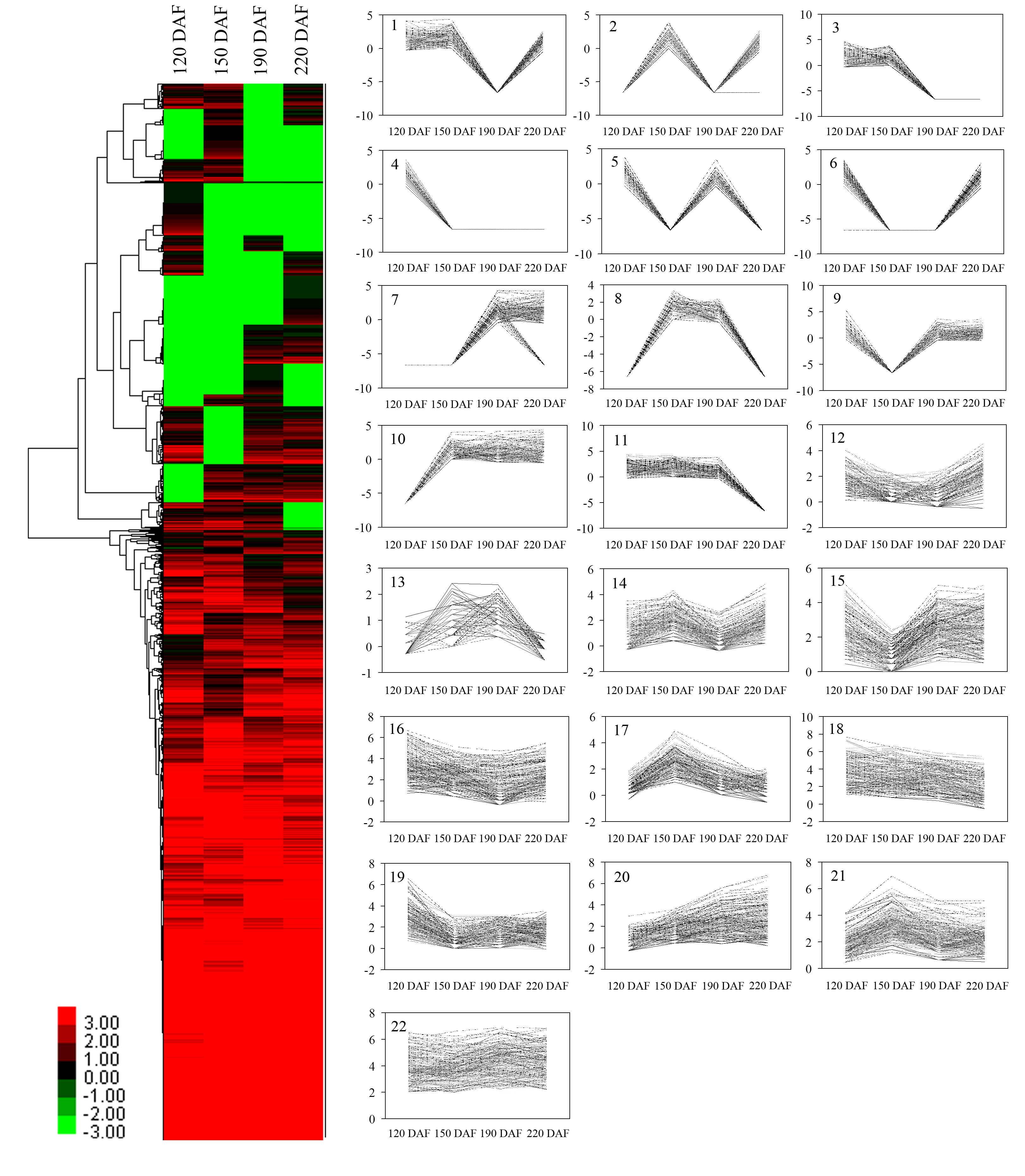
**
