# Supplementary material for: Transcriptome changes during fruit development and ripening of sweet orange (Citrus sinensis)
Source: BMC Genomics. 2012 Jan 10;13:10. doi: 10.1186/1471-2164-13-10 (PMC3267696; doi:10.1186/1471-2164-13-10)
Supplement: Additional file 8 — The ten most differentially expressed genes between MT and WT at each of the four selected fruit developmental stages. This file listed the ten most differentially expressed genes between MT and WT at different developmental stages, with their expression ratios between MT and WT, also containing simple annotation information. [file 1471-2164-13-10-S8.DOC]

**Additional file 8 The ten most differentially expressed genes between MT and WT at each of the four selected fruit developmental stages.**

|  |  |  | **Up-Down-** |  |  |
| --- | --- | --- | --- | --- | --- |
|  | **Gene** | **log2 Ratio** | **regulation** | **P-Value** | **Assignment** |
|  |  | **(MT/WT)** | **(MT/WT)** |  |  |
| 120DAF | TC23713 | 6.882643 | Up | 6.16E-06 | AGAP005067-PA |
|  | TC25336 | 6.6438562 | Up | 1.36E-05 | Heat shock protein 60 |
|  | EY661344 | -5.184996 | Down | 4.32E-05 | Translationally-controlled tumor protein homolog |
|  | TC21043 | 6.2667865 | Up | 4.48E-05 | RNA-directed DNA polymerase |
|  | TC5370 | -5.087463 | Down | 5.95E-05 | Cysteine protease Cp5 |
|  | TC7029 | -4.857981 | Down | 0.00011 | Ribosomal protein S4 |
|  | TC18738 | 5.7548875 | Up | 0.0002 | Expansin |
|  | TC21389 | 5.6147098 | Up | 0.0003 | Latex cyanogenic beta glucosidase |
|  | TC17679 | 5.523562 | Up | 0.00038 | Predicted protein |
|  | TC14207 | -4.087463 | Down | 0.00087 | Elongation factor 1-alpha |
| 150DAF | TC5370 | 6.33985 | Up | 0 | Cysteine protease Cp5 |
|  | EY661344 | 4.7938959 | Up | 1.64E-14 | Translationally-controlled tumor protein homolog |
|  | TC12069 | -5 | Down | 1.19E-12 | Cu/Zn superoxide dismutase |
|  | CB292347 | 3.2302976 | Up | 1.72E-07 | Metallothionein-like protein |
|  | TC271 | -4.169925 | Down | 5.01E-07 | Os12g0506700 protein |
|  | TC10841 | 3.7004397 | Up | 1.07E-05 | S-adenosylmethionine synthetase |
|  | TC3635 | -2.83289 | Down | 1.97E-05 | 18S ribosomal RNA gene |
|  | TC15414 | 2.5501971 | Up | 3.15E-05 | Hydroxyproline-rich glycoprotein precursor |
|  | TC8708 | -2.754888 | Down | 3.39E-05 | Alcohol dehydrogenase |
|  | EY685059 | -3.70044 | Down | 6.15E-05 | SKP1 component-like 1 |
| 190DAF | TC5370 | -6.209453 | Down | 2.42E-12 | Cysteine protease Cp5 |
|  | TC5159 | -5.882643 | Down | 2.93E-11 | Beta-1,3-glucanase class III |
|  | EY732344 | -4.754888 | Down | 6.15E-08 | 45S pre rRNA gene |
|  | EY731511 | -4.276124 | Down | 1.01E-06 | Eukaryotic translation initiation factor 4 gamma 3 |
|  | TC14251 | 4.6438562 | Up | 1.04E-06 | Cytochrome P450 71D8 |
|  | EY733380 | -4.169925 | Down | 1.81E-06 | Plus agglutinin |
|  | EY757336 | 4.5849625 | Up | 1.27E-05 | Hypersensitive-induced response protein |
|  | TC16377 | -4.321928 | Down | 1.44E-05 | Metallothionein-like protein |
|  | TC18565 | -4.247928 | Down | 1.97E-05 | Late embryogenesis abundant protein LEA5 |
|  | EY733268 | -3.857981 | Down | 2.05E-05 | LigA |
| 220DAF | TC5159 | -7.451211 | Down | 1.12E-25 | Beta-1,3-glucanase class III |
|  | TC5370 | -6.442943 | Down | 1.43E-19 | Cysteine protease Cp5 |
|  | TC14251 | 4.357552 | Up | 4.93E-10 | Cytochrome P450 71D8 |
|  | TC2562 | 3.7441611 | Up | 8.60E-08 | Cys-3-His zinc finger protein |
|  | TC1535 | -3.826548 | Down | 8.77E-08 | Non-photosynthetic ferredoxin precursor |
|  | TC10250 | -4 | Down | 1.21E-07 | RING finger protein 43 precursor |
|  | TC9021 | -4.169925 | Down | 5.90E-07 | Os01g0201700 protein |
|  | TC15633 | -3.321928 | Down | 3.53E-06 | Fiber protein Fb31 |
|  | EG358231 | -4.087463 | Down | 4.54E-06 | Transcriptional regulator, LysR family protein |
|  | TC14722 | -4.087463 | Down | 4.54E-06 | Phenylalanine ammonia lyase |

DAF, days after flowering.
