# Supplementary material for: Transcriptome changes during fruit development and ripening of sweet orange (Citrus sinensis)
Source: BMC Genomics. 2012 Jan 10;13:10. doi: 10.1186/1471-2164-13-10 (PMC3267696; doi:10.1186/1471-2164-13-10)
Supplement: Additional file 9 — Dynamics patterns of gene expression of a set of genes differentially expressed between MT and WT at each of the four selected fruit developmental stages. This file contained the result of the hierarchical cluster analysis of expression profiles of differentially expressed genes between MT and WT at different developmental stages. The log2 of the ratio between the MT and the WT TPM for each gene was used to perform the cluster analysis. [file 1471-2164-13-10-S9.DOC]

**Additional file 9 Dynamics patterns of gene expression of a set of genes differentially expressed between MT and WT at each of the four selected fruit developmental stages.** The log_2_ of the ratio between the MT and the WT TPM for each gene was used to perform the cluster analysis.

**
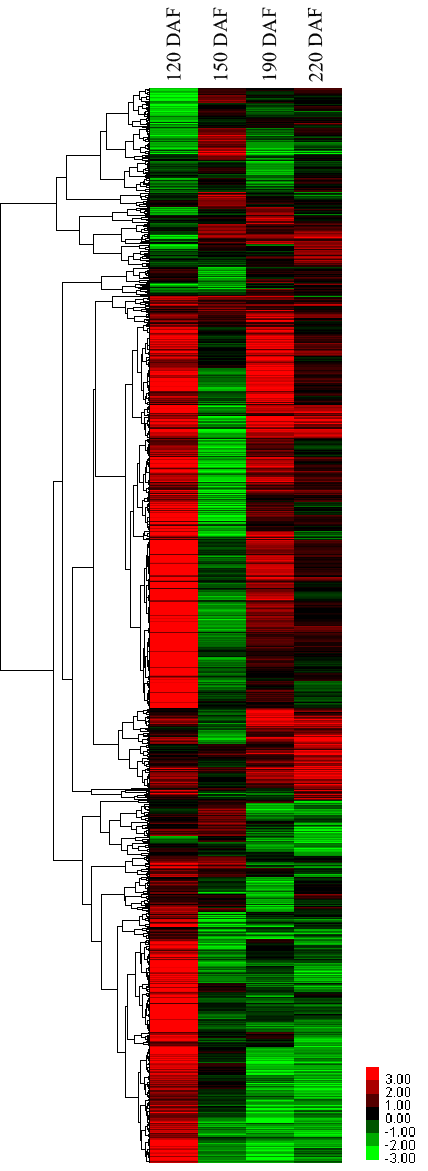
**
