# Supplementary material for: Transcriptome changes during fruit development and ripening of sweet orange (Citrus sinensis)
Source: BMC Genomics. 2012 Jan 10;13:10. doi: 10.1186/1471-2164-13-10 (PMC3267696; doi:10.1186/1471-2164-13-10)

**Additional file 10 The five genes differentially expressed at all four selected developmental stages.** At each stage (120, 150, 190 and 220 DAF), the log_2_ of the ratio between the MT and the WT TPM for each gene is represented. NF, unknown function; NM, no homology in GenBank.


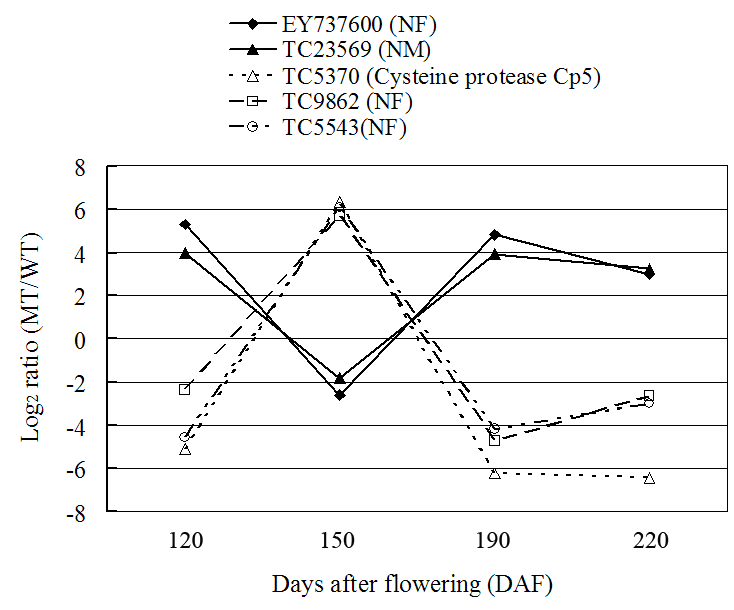

Supplement: Additional file 10 — The five genes differentially expressed at all four selected developmental stages. This file contained the pattern of genes which were differentially expressed at all selected stages. At each stage (120, 150, 190 and 220 DAF), the log2 of the ratio between the MT and the WT TPM for each gene is represented. [file 1471-2164-13-10-S10.DOC]
