# Supplementary material for: Transcriptome changes during fruit development and ripening of sweet orange (Citrus sinensis)
Source: BMC Genomics. 2012 Jan 10;13:10. doi: 10.1186/1471-2164-13-10 (PMC3267696; doi:10.1186/1471-2164-13-10)

**Additional file 11** **Functional categorization of genes differentially expressed between WT and MT at the four selected stages of fruit development and ripening.** The categorization was based on molecular activity of Gene Ontology items. Percentages are based on the proportion of the number of genes in each set.


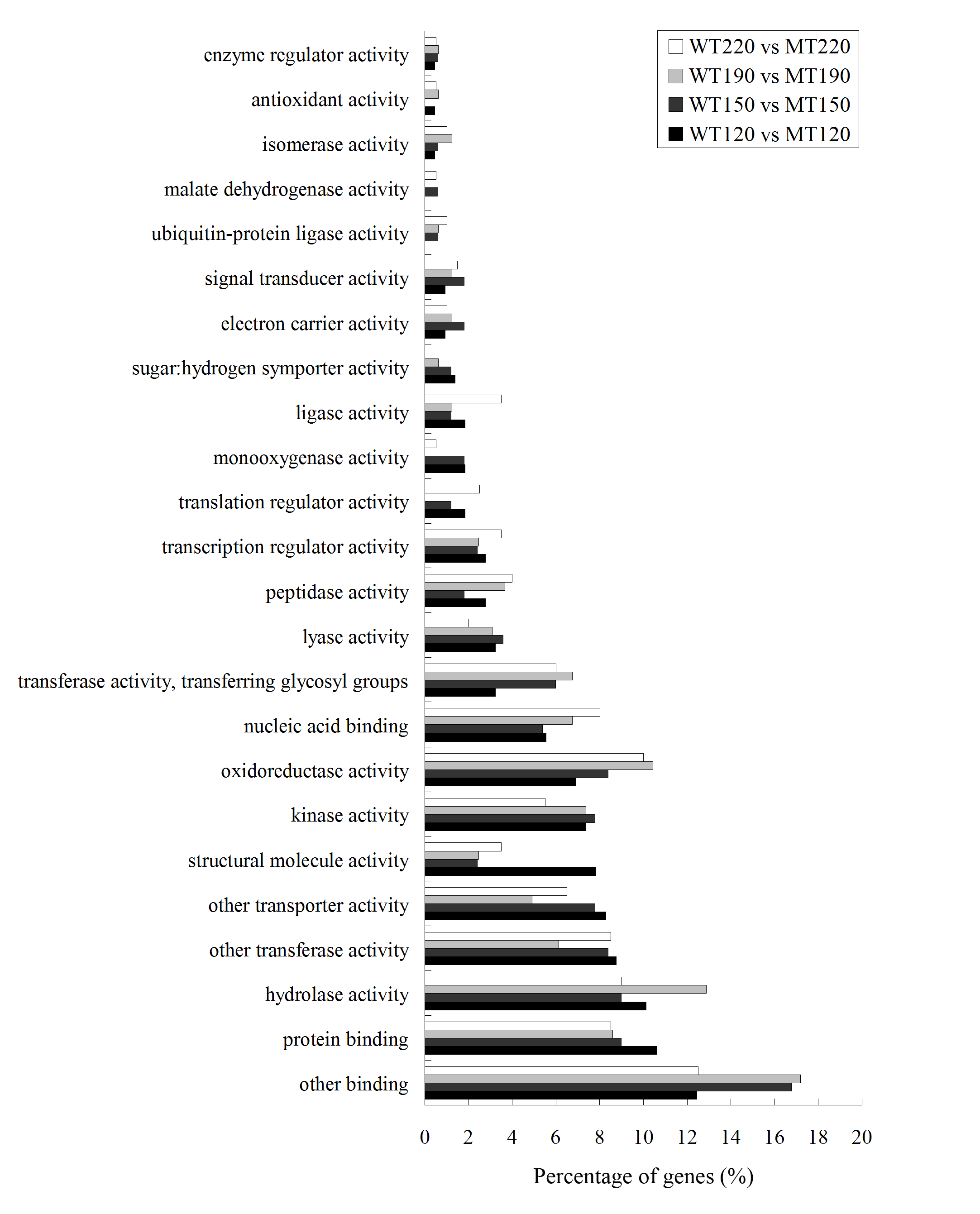

Supplement: Additional file 11 — Functional categorization of genes differentially expressed between WT and MT. This file showed the distribution of GO categories of differentially expressed genes between WT and MT at the four selected stages of fruit development and ripening. The categorization was based on molecular activity of Gene Ontology items. Percentages are based on the proportion of the number of genes in each set. [file 1471-2164-13-10-S11.DOC]
